# Towards High‐Safe Lithium Metal Anodes: Suppressing Lithium Dendrites via Tuning Surface Energy

**DOI:** 10.1002/advs.201600168

**Published:** 2016-07-07

**Authors:** Dong Wang, Wei Zhang, Weitao Zheng, Xiaoqiang Cui, Teófilo Rojo, Qiang Zhang

**Affiliations:** ^1^ Department of Materials ScienceKey Laboratory of Mobile Materials MOEState Key Laboratory of Superhard MaterialsJilin UniversityChangchun130012China; ^2^ IkerbasqueBasque Foundation for ScienceBilbao48011Spain; ^3^ CIC EnergiguneParque Tecnológico de ÁlavaMiñano01510Spain; ^4^ Departamento de Química InorgánicaUniversidad del País VascoUPV/EHU, Bilbao48080Spain; ^5^ Beijing Key Laboratory of Green Chemical Reaction Engineering and TechnologyDepartment of Chemical EngineeringTsinghua UniversityBeijing100084China

**Keywords:** batteries, energy storage, Li‐metal anodes, lithium dendrites, surface energy

## Abstract

The formation of lithium dendrites induces the notorious safety issue and poor cycling life of energy storage devices, such as lithium–sulfur and lithium–air batteries. We propose a surface energy model to describe the complex interface between the lithium anode and electrolyte. A universal strategy of hindering formation of lithium dendrites via tuning surface energy of the relevant thin film growth is suggested. The merit of the novel motif lies not only fundamentally a perfect correlation between electrochemistry and thin film fields, but also significantly promotes larger‐scale application of lithium–sulfur and lithium–air batteries, as well as other metal batteries (e.g., Zn, Na, K, Cu, Ag, and Sn).

## 1 Introduction

**Figure 1:**
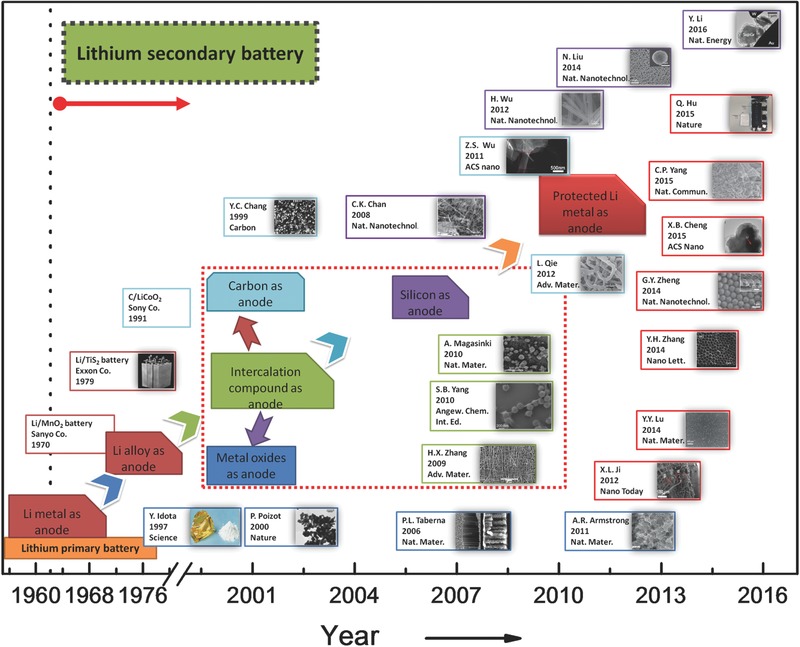
Selected major events of anode in lithium battery from 1955 to 2016. The red imaginary point block diagram represents Li‐metal‐free anode. Sanyo Co., Exxon Co. and Sony Co., referenced in Ref. 48 Reproduced with permission.^48^ Copyright 2004, American Chemical Society. The illustrated box color corresponds to the anode types: Li metal in red; carbon in cyan; metal oxide in blue; silicon in purple; the composite intercalation compound material in green. Idota, tin‐based composite oxide. Reproduced with permission.^49^ Copyright 1997, American Association for the Advancement of Science. Chang, MCBC (mesophase carbon micro beads) Reproduced with permission.^50^ Copyright 1997, Elsevier. Poizot, nano‐size CoO. Reproduced with permission.^51^ Copyright 2000, Nature Publishing Group. Taberna, Fe_3_O_4_‐based Cu nano‐architectured electrodes. Reproduced with permission.^52^ Copyright 2006, Nature Publishing Group. Chan, silicon nanowires. Reproduced with permission.^53^ Copyright 2008, Nature Publishing Group. Zhang, CNT@SnO_2_, Reproduced with permission.^54^ Yang, graphene@Co_3_O_4_, Reproduced with permission.^55^ Magasinski, C–Si nanocomposite, Reproduced with permission.^56^ Copyright 2010, Nature Publishing Group. Wu, doped graphene sheets. Reproduced with permission.^57^ Copyright 2011, American Chemical Society. Armstrong, Li_1+_ *_x_*V_1–_ *_x_*O_2_. Reproduced with permission.^58^ Copyright 2011, Nature Publishing Group. Wu, double‐walled silicon nanotube, Reproduced with permission.^59^ Copyright 2012, Nature Publishing Group. Ji, Li metal on carbon‐fiber papers. Reproduced with permission.^60^ Copyright 2012, Elsevier. Qie, Nitrogen‐doped porous carbon nanofiber webs. Reproduced with permission.^61^ Liu, silicon nanoparticles. Reproduced with permission.[[qv: 46b]] Copyright 2014, Nature Publishing Group. Lu, LiF cluster on Li foil. Reproduced with permission.[[qv: 14b]] Copyright 2014, Nature Publishing Group. Zhang, Li deposition in Cs^+^‐containing electrolyte. Reproduced with permission.^27^ Copyright 2014, American Chemical Society. Zheng, interconnected hollow carbon nanospheres. Reproduced with permission.[[qv: 18a]] Copyright 2014, Nature Publishing Group. Yang, 3D porous Cu foil. Reproduced with permission.^44^ Copyright 2015, Nature Publishing Group. Hu, a SolidEnergy prototype battery with ultra‐thin Li metal anode. Reproduced with permission.^62^ Copyright 2015, Nature Publishing Group. Li, conformal graphene cages on micrometre‐sized silicon particles. Reproduced with permission.^63^ Copyright 2016, Nature Publishing Group.

The last century has witnessed the soaring development of Li primary batteries (**Figure** **1**). The concept of Li second battery was initialized in 1962.^1^ However, the notorious safety issue of Li metal retards its worldwide commercialization. Since the rechargeable Li‐ion batteries with graphite anodes were announced in the early 1990s,^2^ great success has been achieved for portable devices and electric vehicles. However, the demand in energy density is increasing. Exploring portable energy storage devices with high energy density is strongly considered. Both Li–S batteries^3^ and Li–O_2_ batteries are believed as the next‐generation energy storage systems with ultrahigh energy density.^4^ Li metal anode is employed in these emerging systems. Aimed at an energy capacity beyond the practical 500 Wh/kg, the Li–S battery has attracted wide interests. In spite of rapid development in sulfur cathode, Li metal anode remains a main challenge for bulk application.^5^ Recently, more importance has been attached to the protection of Li metal anode, as summarized in **Table** **1**.

**Table 1:** Timeline of Li metal anode

Time	Milestone	Research group	Remarks	Refs.
1910∼1920	The initial study of lithium battery	Lewis	Li metal as electrode	68
1962	The rising Li secondary battery	Whittingham	Li secondary battery in non‐aqueous solution	[[qv: 1a]]
1970s	First commercialization of Li metal battery	Sanyo Co.	Lithium primary battery	48
1976	Discovery of Li ions embed into the carbon	Besenhard Agarwal	Li metal anode neglected gradually in secondary battery	69
1983	The development of cathode materials for Li‐ion battery	Goodenough		70
1991	First commercialization of Li ion battery	Sony Co.	Li metal free anode emerged	48
2000∼2010	Searching for high energy density battery	Dudney SionPower Co. PolyPlus Co.	The efforts to solve the problem of Li metal anode	[[qv: 19b,20,23,71]]
2009	The re‐emerging Li‐S battery attention	Nazar	The interest of Li metal anode was refueled	3
2012	Carbon‐fiber papers as surface dendrite‐free current collector for lithium deposition	Stucky		60
2013	Dendrite‐free lithium deposition *via* self‐healing electrostatic shield model	Zhang		12, 27
2013	Solvent‐in‐Salt electrolyte	Hu		[75]
2014	Protecting Li anode though SEI film	Archer		[[qv: 14b,72]]
2014	Protecting Li anode though film deposition	Cui		18
2014	Li anode protection though	Wen		25
	LiN_3_ SEI layer coating			
2015–2016	Li metal protection though 3D graphene and oxide electrolyte	Zhang		[[qv: 10a,14a,26a]]
2015–2016	Protecting Li anode through 3D Cu current collector or Li_3_PO_4_ SEI layer coating	Guo		[[qv: 26b,44]]

**Figure 2:**
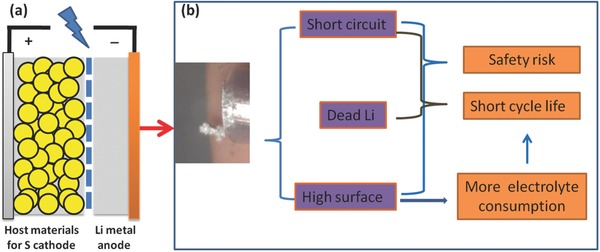
Schematic diagram of a) Li–S batteries with Li metal as anode; b) the typical morphology of Li dendrites and the main issues related to the dendrites (see Crowther and West, Reproduced with permission.[[qv: 21a]] Copyright 2008, The Electrochemical Society..

Among various rechargeable batteries with high energy density and long cycle life, both Li–S and Li–O_2_ batteries are strongly considered, ascribed to the large theoretical specific energy densities (≈2600 and ≈11400 Wh kg^–1^ for Li–S and Li–O_2_ batteries, respectively), with an order of magnitude higher than that of Li‐ion battery (e.g., ≈360 Wh kg^–1^ for LiCo_2_O_4_/C). The Li metal renders an extremely high theoretical specific capacity (3860 mA hg^–1^), a very low negative redox potential (‐3.040V vs standard hydrogen electrode), and a low density (0.59 g cm^–3^). Unfortunately, there are still two important issues to be addressed: on one hand, the breaking of the formed Li dendrites (**Figure** **2**b) induces the so‐called “dead Li” (rarely contributing to the battery capacity); on the other hand, the continuous consumption of the electrolyte resulted from the high reactivity of fresh Li surface. In addition, the explosion of Li metal anode, originated from short circuit associated with Li dendrites, also raises a risk to Li metal battery. Therefore, it is critical to overcome these technological challenges of Li metal anodes in order to unlock the full potential in achieving the theoretical performance of Li metal batteries.^6^ One of the essential central issues is to probe an accurate and universal understanding of the formation mechanism and blocking strategy of Li dendrites. Meanwhile, there are more scientific issues to be considered for the whole working battery. For instance, although polysulfides diffused from the sulfur cathode has been evidenced that it can sustain the Li dendrite growth in a Li–S cell,^7^ more other severe electrochemical challenges (including Li anode protection at high current densities) are triggered (Figure 2a).^8^

This is an open access article under the terms of the Creative Commons Attribution License, which permits use, distribution and reproduction in any medium, provided the original work is properly cited.

## 2 Models and Mechanism of Li Dendrites

Several theoretical models have been proposed to describe formation and growth behaviors of Li dendrites, including phase‐field model,^9^ solid electrolyte interphase (SEI) model,^10^ deposition/dissolution model,^11^ and charge induced growth model.^12^ As shown in **Table** **2**, several aspects can be clarified. These models are catalogued into the following two basic modes (rooted from which phase‐field model is a mathematical simulation calculation) from a point view of the dendrite formation.

1) Models in thermodynamics mode, that is, deposition/dissolution model^11^

a) Li metal is deposited beneath the SEI film.
b) Supplied with an external power, Li ions in the electrolyte transport to Li metal surface through the protective SEI film. The deposition sites on the protective film exhibit a higher Li^+^ conductivity. As a result, crystal defects and grain boundaries in the SEI initiate the continuous deposition of Li.
c) The mechanical stress within the Li metal anode induces an asymmetrical deposition of Li, resulting in the formation of Li dendrites.

**Table 2:** Various models for Li dendrite/protection

Models	Mechanisms	Merit	Refs.
Phase‐field model	Mathematics model for Li dendrite	Wide application for calculation of the key events of kinetics	9, 73
SEI model	Electrochemistry model for Li protection	Widely accepted in efforts to form a matching SEI film in order to protect Li	[[qv: 10b,14b,18,20,22,24,25,37b,46a,74]]
Charge‐based model	Electrochemistry model for Li protection/dendrite	Application leading to Li dendrite free cases	12, 27
Deposition and dissolution model	Thermodynamics model for Li dendrite	Well accepted to understand the Li dendrite behaviors	11
Film growth model	Thermodynamics model for Li dendrite/Li protection	Inspired by growth behaviors of special nanocarbon forms to reinvent our understanding of the dendrite issues	This work

Supposing that lithium dendrite stems from the mechanical stress, the following Laplace's Equation (1) applies in this model based on the droplet theory of homogeneous nucleation, to be detailed in Section 4:

(1) $$\Delta P=\gamma \left(1/R_{1}+1/R_{2}\right)$$

where Δ*P* is the pressure difference of the surface, γ is the surface tension on a lithium surface, and *R*_1_ and *R*_2_ are any two orthogonal direction radius of the surface curvature. Yamaki et al. set out the dendrites form because of the surface tension effects, and proposed that the surface tension of protective film must be higher than 0.2 Nm^–1^ by simulation.^11^ On the base of this model, a substantial film coating on Li metal can block the Li dendrite obviously.

2) Charge induced growth model^12^ (one of the electrochemistry models)

Li dendrite growth is considered the adoption of one dimensional nanostructures growth by electrodeposition.^13^ Through applying the charge induced growth model, as the Li ions are deposited in combination with the surface charge, the inhomogeneous charge distribution leads to the Li dendrite. This model is on the basis of the Nernst Equation (2):

(2) $$\;E_{\mathrm{Red}}=\;E_{\mathrm{Red}}^{\phi }-\frac{RT}{nF}\ln\frac{\alpha_{\mathrm{Red}}}{\alpha_{Ox}}$$

where *E*_Red_ is a reduction potential ($E_{\mathrm{Red}}^{\phi }$ is the standard reduction potential), *R* is the universal gas constant (8.31 J K^–1^ mol^–1^), *T* is the absolute temperature, *α* is the chemical activity for the relevant species (*α*_Red_ is for the reductant and *α*_Ox_ is for the oxidant), *F* is the Faraday constant (9.65 × 10^4^ C mol^–1^), and *n* is the number of transferred electrons. On the basis of this model, another metal cation (M^+^) may have an effective reduced potential lower than that of Li^+^ if M^+^ has a chemical activity α lower than that of Li^+^. Consequently, it indicates that the electrolyte additive cation should have an effective reduction potential lower than that of Li ion. Although this model does not unravel the dendrite origin, it provides a roadmap to a self‐healing electrostatic shield to make the dendrite free.

3) Models in kinetics mode, e.g., SEI model (one of the electrochemistry models)^14^

The composition of SEI model is highly dependent on the voltage and history, correlated closely with the thermodynamics mode mentioned above. For instance, by applying SEI model on the basis of the thermodynamics mode, the way for protecting Li anode lies in that we should find a high‐modulus electrolyte.[[qv: 14b]] The kinetics mode, however, can be also intermediated, although the linkage is usually underestimated.

Several kinetics models have been proposed to describe the Li dendrite formation. The occurrence of Li ions near the Li metal anode results from the charge at a high current density, as well as the drain of Li salt anions in Sand's time.^15^ As a consequence, the lack of Li^+^ layer, coupled with the local space charge layer, is regarded as the main reason for the formation of Li dendrite. Together with many efforts made for this theory based on kinetics^16^ aspects of Li dendrites, Chazalviel^15^, ^17^ proposed the widely accepted diffusion model (Equation (3)) to correlate the “Sand's time” *τ* with the transfer nature of Li^+^ ions and electrons empirically as follows,

(3) $$\tau \;=\;\pi D\frac{eC_{0}{\left(\mu_{a}+\;\mu_{{\mathrm{Li}}^{+}}\right)}^{2}}{2J\mu_{a}}$$

where *τ* is the initial time of Li dendrites growth, *C*_0_ is the initial concentration of Li salt, *D* is the diffusion coefficient, *e* is the electronic charge, and *J* is the effective electrode current density. *μ*_a_ and $\mu_{{\mathrm{Li}}^{+}}$ are the mobilities of anionic and Li^+^, respectively. Hence the SEI region can be also treated as a kinetics‐controlling area, on the condition that the SEI film locates within the thermodynamic stability. It is well accepted that a suitable SEI film is obtained to suppress the dendrite by affecting $\mu_{{\mathrm{Li}}^{+}}$ in governing the Sand's time (*τ*). Meanwhile, the effective electrode current density (*J*) is correlated with the transport of electrons, leading to dendrite‐free anodes. This theory model can largely strengthen our understanding of the Li dendrite mechanism, which enables a long‐life and safe Li anode through the application of SEI film regulation[[qv: 10c]] or an ultralow current density.[[qv: 14a]]

Although the merit of various models have been revealed the formation of Li dendrites, and they are complementary with each other, it is necessary to establish a general model in unraveling the thermodynamic and kinetic aspects of Li dendrites. In fact, the substrate (Li or other materials) and the SEI region (considered from Chazalviel theory) depend partially on the thermodynamics and kinetics factors during the plating, respectively. Herein we describe a film growth model in order to provide fresh insight into the Li dendrite with focusing on the thermodynamics aspect of Li dendrite. Moreover, we demonstrate that it enables an even more thorough understanding of Li dendrites and thereof an effective strategy is established in facilitating Li metal anode protection.

## 3 Exploration for Li Metal Protection

**Figure 3:**
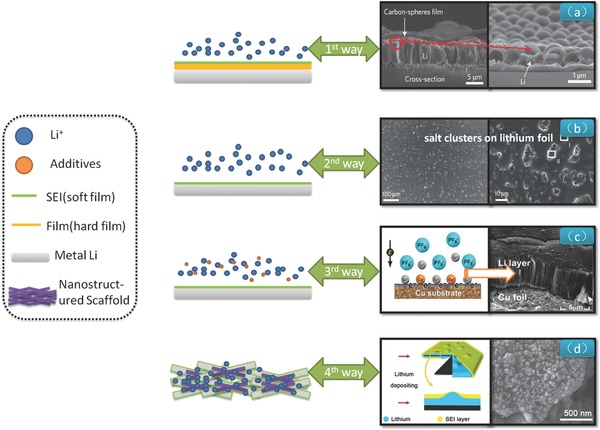
Schematic diagram of four ways for Li metal protection. From 1st to 4th ways in order, 1) the film of interconnected hollow carbon spheres isolated the Li metal and electrolyte. Reproduced with permission.[[qv: 18a]] Copyright 2014, Nature Publishing Group; 2) SEI film composed by LiF coated Li foil. Reproduced with permission.[[qv: 14b]] Copyright 2014, Nature Publishing Group; 3) the dendrite‐free Li deposition by Cs^+^ addition at 0.1 mAh cm^–2^ for 15 h. Reproduced with permission.^27^ Copyright 2014, American Chemical Society; 4) the graphene‐based conductive nanostructured scaffolds anode leads to a low local current density for Li plating. Reproduced with permission.[[qv: 14a]]

The soaring progress of Li metal batteries drives scientific communities to renaissance the Li metal protection. The main technological routes have been established (**Figure** **3**):

(1) A "hard film" is prepared to block the Li dendrites.^18^
(2) Li is promoted to react with other materials^19^ in order to form a targeting "soft film", such as SEI film for the suppression of the Li dendrite growth.
(3) Additives are introduced to electrolyte to postpone/retard the dendrite growth^20^ or even get a dendrite‐free Li anode.^21^
(4) The use of nanostructure to modulate the Li deposition behavior through ultralow current density.[[qv: 14a]]

Although many efforts have been devoted to reduce the Li dendrites, there is a long way for the ultimate solving towards the protection of the Li metal anode. This is particularly critical for cycling of Li metal anode at very high current density. One representative work of “hard film” coating was proposed by Cui's group.[[qv: 18a]] The thin film with high ionic conductivity and high Young's modulus is introduced to the Li anode in order to block the Li dendrites and prevent the Li metal to immediately contact the electrolyte.

In terms of SEI film, Li salt mixtures offer a thin passivation layer which could suppress the formation of Li dendrites by the Archer's group.[[qv: 14b,22]] However, it is very difficult to form a stable passivation film on Li electrodes,^23^ due to the thermodynamically unstable of Li metal in organic solvents. Therefore, the continuing target is to search for a matching SEI film[[qv: 10a,24]] or Li salt film.^20^, ^25^ Cheng et al.^26^ propose the efficient use of stable solid electrolyte interphase in a high‐efficiency battery in order to block the formation of Li dendrites. Alternatively, some additives can be introduced. Zhang and co‐workers^27^ reported that a dendrite‐free lithium deposition was successfully achieved with the self‐aligned nanorod structure through adding Cs^+^. The consumption of the electrolyte suffer from the immediate contacting between Li anode and electrolyte. An interesting work by Kim and co‐workers[[qv: 21b]] illustrates that Li dendrites are controlled via a synergistic effect of multilayered graphene coating and an Cs^+^ electrolyte additive. The use of nanostructured framework with high surface area and extraordinary conductivity renders a very low local current together with robust SEI in dual‐salt organic electrolyte afford the smooth and continuous Li deposition without dendrite formation.[[qv: 14a,28]]

## 4 Film Growth Model

**Figure 4:**
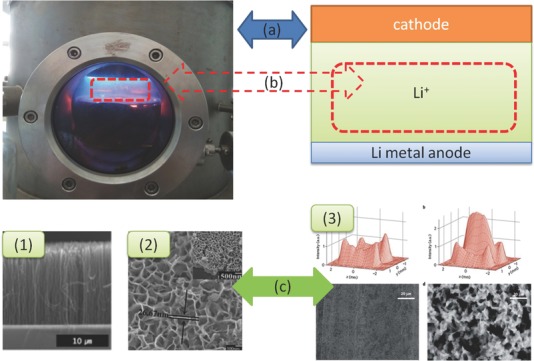
Schematic illustrations of vertical materials grown by PECVD and the dendrites in Li metal batteries, there are similarities a) in the electric filed (radio frequency electric filed in PECVD, the potential of electric filed in batteries), b) with the active materials, and c) for the final product: 1) carbon nanotubes. Reproduced with permission.^64^ 2) vertical graphene sheets. Reproduced with permission.^65^ Copyright 2014, Elsevier; 3) Li dendrites. Reproduced with permission.^66^ Copyright 2012, Nature Publishing Group.

The growth of Li dendrites belong to a typical crystal growth in an organic solution.^29^ It is well known that the Volbmer–Weber mode for growth of polycrystalline films, which comprises the island, network, and channel stages by CVD^30^ as well as particularly for plasma enhanced chemical vapor deposition (PECVD)^31^ towards vertically grown materials under the condition of an electric field. As shown in **Figure** **4**, since PECVD offers an electric field,^32^ a vertical film on substrate is achieved from the cooperation of the active species (e.g., the fragment of CH_4_ and CH_3_^+^) and transition metal catalyst^33^ (e.g., Fe, Co, Ni). The resultant products can be the vertical carbon nanotubes (Figure 4), vertical graphene sheet (Figure 4). Inspired by the PECVD advantages, in analogy to the aforementioned process, the active species (Li^+^) and catalyst (the charge accumulation area), under the external electric field, work together, leading to the formation of Li dendrites (Figure 4). The formation and continuous growth of Li dendrites is attributed to the following scenarios: the Li^+^ ions near the anode, promoted under the electric filed, induces Li deposition on the anode; as a consequence, an increase of surface energy occurs. We call it the *film growth* model and explore the dendrite issue by applying classical theory of film within a battery system later.

**Figure 5:**
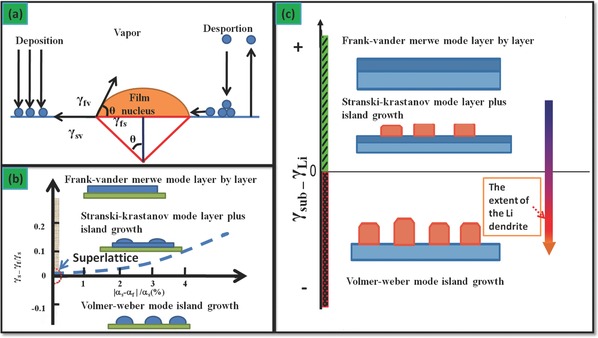
a) Scheme of basic atomistic nucleation on substrate surface during vapor deposition;^67^ b) The stability regions of the three‐film growth modes in coordination of surface energy differences between growing film and growth substrate (vertical)/the lattice misfit (horizontal);^67^ a,b) redrawn after the reference. c) Scheme of correlation between the extent of Li dendrites and the surface energy difference.

Our film growth model is to address the Li dendrite issues via the surface energy^30^, ^34^ since the latter can be listed as one of the film growth. Based on the well‐known capillarity of homogeneous nucleation, a solid nucleates from a prior unstable liquid by establishing a solid–liquid (s–l) interface; in analogue for the droplet theory of homogeneous nucleation (the deposition/dissolution model stem from this theory), a solid nucleates from vapor phase by establishing a solid–vapor (s–v) interface. These two scenarios are depicted as **Figure** **5**A.

According to the Young's equation,

(4) $$\;\gamma_{sv\;}=\;\gamma_{fs}+\;\gamma_{fy}\cos\theta$$

where *f*, *s*, and *v* denote the film, the substrate and the vapor, respectively. Then *γ_sv_*, *γ_fs_*, *γ_fv_* represent the interface energy between the two phases, *θ* is the contact angle. Based on Equation (4), it is well accepted that the film growth depends on two main issues: 1) the surface energy difference between the substrate and the film; 2) the lattice misfit between growth substrate and growing film, as shown in Figure 5B.

**Figure 6:**
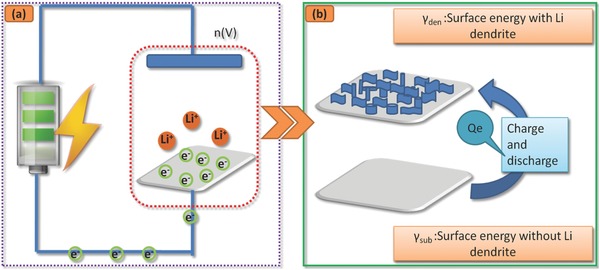
Schematic diagram of Li dendrites a) describes that plenty of charge around the Li anode surface during an external power supply, b) shows the surface topography become rough, ascribed to the change of surface energy during the charging/discharging process).

As shown in Figure 5C, Li deposition is assumed as homogeneous progress of film growth, therefore, the lattice distortion is neglected. With the intermediation of the electric filed, the fluctuation of the surface energy attributed from the dynamics of SEI film induces the Li film growth towards the island type within the framework of Volmer‐Weber mode. As shown in **Figure** **6**A, there are *m* electrons on the anode surface, subjected to an external power supply *n* (V). Consequently, the electrical potential energy can be expressed as *n* × *m* (eV), where *n* is negative. The surface energy of substrate γ_sub_ consists of two terms, the surface energy of a deposition film γ_Li_ and the electrical potential energy. Since γ_sub_ is less than γ_Li_, the Li dendrite is induced if the Li metal is electrodeposited.

Figure 6B exhibits that the Li substrate surface is stable. The surface is free of any Li dendrite and therefore has a constant surface energy γ_sub_. After several charge/discharge cycling, the initialization of the Li dendrites leads to a large surface energy. Concurrently, γ_den_ increases. As a result, the substrate becomes rough. The charge/discharge process suffers from the rise of surface energy, which results in the unfavorable low Coulomb efficiency (CE). As expressed by equation (5),

(5) $$\gamma_{\mathrm{sub}}+Q_{e}=\gamma_{\mathrm{den}}$$

where *Q_e_* stems from the partial energy of the electric field. The larger *Q_e_* becomes, the lower CE changes.

## 5 Roadmaps to Dendrite‐Free Li Anode through our Model

Different from the SEI model mentioned above, we suggest two promising routes to suppress the formation of Li dendrites on the basis of our film growth model derived from the Volmer–Weber theory.^35^

The first direction lies to modulate the surface energy through tuning composition and morphology of Li metal. An excellent Li metal anode is expected through 3D substrates with continuous high surface energy. If *γ*_sub_ increases, the additional barrier is bypassed for the Volmer–Weber mode. As a result, the deposited Li surface becomes smooth, which induces a symmetrical charge/discharge cycling and thereof a higher CE. In parallel, more importance has been practically attached to exploration of Li–alloy anode, which are involved with the intercalation and de‐intercalation of Li in Li metal battery,^36^ LiAl and other Li based alloys.^37^ However, for even higher Li content alloy,^38^ there is a positive effect in Li protection for robust cycling. The metal mixture in anode results in an increase of surface energy,^39^ which promotes a layer growth instead of an island growth. Consequently, the Li dendrite formation is postponed or even completely suppressed.

**Figure 7:**
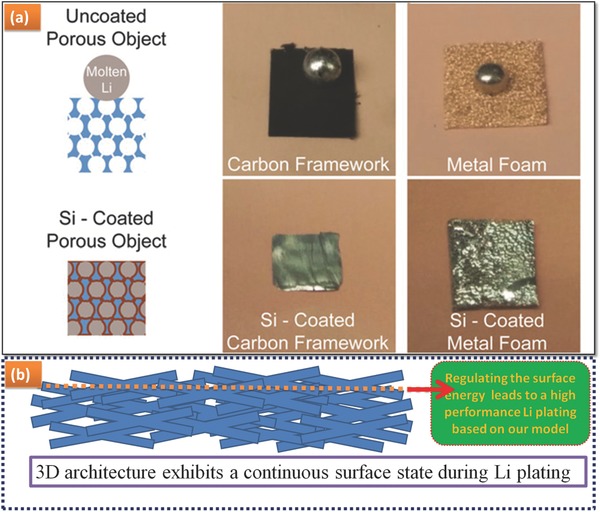
a) Schematic diagram of surface‐modified 3D substrate with a “lithiophilic” coating. Reproduced with permission.[[qv: 43a]] Copyright 2016, National Academy of Sciences; b) Schematic diagram of our roadmap: a continuous surface possessing high surface energy.

In order to avoid the occurrence of potential active sites for Li dendrite nucleates, the Li metal surface should be smooth.^40^ Recently, both Li powder^41^ and Li foam^42^ anode are treated as a positive way to suppress Li dendrite formation, as it is common knowledge that the nanostructured counterpart has a larger surface area and surface energy. This can be well explained by our surface energy scenarios. That is, more importance should be attached to controlling and governing of the substrate in Li deposition, e.g., the Cui's latest work.^43^ Specifically, Guo's group successfully suppressed Li dendrite^44^ by applying a 3D current collector, explained by the electric field effect as anode substrate. The use of 3D porous Cu exhibits a high surface energy, and thereof the mechanism is factually explained by the tuning of surface energy and film growth model. Layer‐by‐layer growth Li film induces a few dead‐Li system, resulting in a long‐life anode, as shown in Figure 1. Modulating the anode surface with high surface energy is the very roadmap to obtain a dendrite‐free Li metal anode. Very recently, the research work (**Figure** **7**a) of surface‐modified three‐dimensional (3D) substrate with a “lithiophilic” coating,[[qv: 43a]] presents a novel strategy for the fabrication of metal–scaffold composite. Specifically, the resultant material was employed as anodes in Li metal batteries, exhibiting superior performance compared with bare lithium metal anodes. On the basis of our model, the surface energy (in J cm^–2^ or eV cm^–2^ for the surface perpendicular to the electric field during the Li plating) of 3D substrate yields a continuous surface state, as shown in Figure 7b. A long‐life and safe Li metal anode can be fabricated.

The second way is reducing the electrical potential energy *n* × *m* (eV), which contributes to the decrease of the term γ_sub_–γ_Li_. According to our model, the Li dendrites enable being thereof suppressed. This event is achieved by altering the charge mode towards tuning the electrical potential energy, of which the application of pulse charging^45^ or charging at a lower voltage would be some of the best choices. The pulse charging mode would lead to a process of relaxation surface charge, and tailoring of the electrical potential energy. The use of gel electrolytes and polarized framework is another route to tune the transport and nucleate behavior of Li ions onto Li metal anode.[[qv: 10a,46]] The use of high current is also verified to protect the robust use of Li metal anode.^47^

## 6 Concluding Remarks

The safe use of Li metal is strongly considered for high energy batteries. We introduced the concept of surface energy to understand to the formation and growth of Li dendrites in Li metal batteries. A high charge/discharge current is required if the increased surface energy cannot override the effect of electric field, alternatively a hard film is required to block the Li dendrites. However, the energy relaxation changes to the thermal energy, which is a risk for a working cell. In addition, the proposed model is themed on the effect of thermodynamics, which is the emerging supplement to the kinetics model (SEI model analyzed by Chazalviel theory). The way addressing the kinetics effects focus on the homogenous surface (a robust and homogenous SEI film), whereas the way addressing the thermodynamic effects should gear to the continuous surface (the state of substrate). Consequently, the golden rule lies on both of them for Li protection.

Li anode protection doesn't barely regulate to dendrite‐free Li metal anodes, the problem of electrolyte consumption should be addressed to avoid an unexpected reaction between Li and electrolyte. A continuous surface possessing high surface energy during the charge/discharge cycling is promising to suppress the lithium dendrites. Such concept may be extended to other metal batteries, such as Zn, Na, K, Cu, Ag and Sn.

## References

[1] a) R. Osteryoung , R. McKisson , P. Dutch , G. Lauer , E. Luchsinger , Technical Reports, AD‐290326, Atomics International Div, Canoga Park, California, 1962;

[2] T. Nagaura , presented at 4th Int. Rechargeable Battery Seminar, Deerfield Beach, FL, USA, 1990.

[3] X. Ji , K. T. Lee , L. F. Nazar , Nat. Mater. 2009, 8, 500. 19448613 10.1038/nmat2460

[4] L. Grande , E. Paillard , J. Hassoun , J.‐B. Park , Y.‐J. Lee , Y.‐K. Sun , S. Passerini , B. Scrosati , Adv. Mater. 2015, 27, 784. 25645073 10.1002/adma.201403064

[5] J. W. Choi , D. Aurbach , Nat. Rev. Mater. 2016, 1, 16013.

[6] a) R. Cao , W. Xu , D. Lv , J. Xiao , J.‐G. Zhang , Adv. Energy Mater. 2015, 5, 1402273;

[7] W. Li , H. Yao , K. Yan , G. Zheng , Z. Liang , Y.‐M. Chiang , Y. Cui , Nat. Commun. 2015, 6, 7436. 26081242 10.1038/ncomms8436

[8] a) C. Zu , A. Manthiram , J. Phys. Chem. Lett. 2014, 5, 2522; 26277939 10.1021/jz501352e

[9] Y. Okajima , Y. Shibuta , T. Suzuki , Comp. Mater. Sci. 2010, 50, 118.

[10] a) X.‐B. Cheng , T.‐Z. Hou , R. Zhang , H.‐J. Peng , C.‐Z. Zhao , J.‐Q. Huang , Q. Zhang , Adv. Mater. 2016, 28, 2888; 26900679 10.1002/adma.201506124

[11] J.‐i. Yamaki , S.‐i. Tobishima , K. Hayashi , S. Keiichi , Y. Nemoto , M. Arakawa , J. Power Sources 1998, 74, 219.

[12] F. Ding , W. Xu , G. L. Graff , J. Zhang , M. L. Sushko , X. Chen , Y. Shao , M. H. Engelhard , Z. Nie , J. Xiao , X. Liu , P. V. Sushko , J. Liu , J.‐G. Zhang , J. Am. Chem. Soc. 2013, 135, 4450. 23448508 10.1021/ja312241y

[13] C. D. Owen , M. Grant Norton , J. Mater. Sci. 2016, 51, 577.

[14] a) R. Zhang , X.‐B. Cheng , C.‐Z. Zhao , H.‐J. Peng , J.‐L. Shi , J.‐Q. Huang , J. Wang , F. Wei , Q. Zhang , Adv. Mater. 2016, 28, 2155; 26754639 10.1002/adma.201504117

[15] J. N. Chazalviel , Phys. Rev. A 1990, 42, 7355. 9904050 10.1103/physreva.42.7355

[16] a) C. Monroe , J. Newman , J. Electrochem. Soc. 2005, 152, A396;

[17] C. Brissot , M. Rosso , J. N. Chazalviel , P. Baudry , S. Lascaud , Electrochim. Acta 1998, 43, 1569.

[18] a) G. Zheng , S. W. Lee , Z. Liang , H.‐W. Lee , K. Yan , H. Yao , H. Wang , W. Li , S. Chu , Y. Cui , Nat. Nano 2014, 9, 618; 10.1038/nnano.2014.152 25064396

[19] a) J. B. Bates (Martin Marietta Energy Systems, Inc, Oak Ridge, TN, USA) *US patent, 5, 314, 765*, 1994;

[20] S. S. Zhang , Electrochim. Acta 2012, 70, 344.

[21] a) O. Crowther , A. C. West , J. Electrochem. Soc. 2008, 155, A806;

[22] Z. Tu , Y. Lu , L. Archer , Small 2015, 11, 2631. 25677882 10.1002/smll.201403568

[23] D. Aurbach , E. Zinigrad , Y. Cohen , H. Teller , Solid State Ionics 2002, 148, 405.

[24] K. Xu , Chem. Rev. 2014, 114, 11503. 25351820 10.1021/cr500003w

[25] G. Ma , Z. Wen , M. Wu , C. Chen , Q. Wang , J. Jin , X. Wu , Chem. Comm. 2014, 50, 14209. 25285341 10.1039/c4cc05535g

[26] a) X.‐B. Cheng , H.‐J. Peng , J.‐Q. Huang , R. Zhang , C.‐Z. Zhao , Q. Zhang , ACS Nano 2015, 9, 6373; 26042545 10.1021/acsnano.5b01990

[27] Y. Zhang , J. Qian , W. Xu , S. M. Russell , X. Chen , E. Nasybulin , P. Bhattacharya , M. H. Engelhard , D. Mei , R. Cao , F. Ding , A. V. Cresce , K. Xu , J.‐G. Zhang , Nano Lett. 2014, 14, 6889. 25419865 10.1021/nl5039117

[28] R. Miao , J. Yang , X. Feng , H. Jia , J. Wang , Y. Nuli , J. Power Sources 2014, 271, 291.

[29] W. Ye , C. Shen , J. Tian , C. Wang , L. Bao , H. Gao , Electrochem. Comm. 2008, 10, 625.

[30] Y. Kajikawa , S. Noda , Appl. Surf. Sci. 2005, 245, 281.

[31] a) Z. Bo , Y. Yang , J. Chen , K. Yu , J. Yan , K. Cen , Nanoscale 2013, 5, 5180; 23670071 10.1039/c3nr33449j

[32] Y. Zhang , A. Chang , J. Cao , Q. Wang , W. Kim , Y. Li , N. Morris , E. Yenilmez , J. Kong , H. Dai , Appl. Phys. Lett. 2001, 79, 3155.

[33] C. Ducati , I. Alexandrou , M. Chhowalla , J. Robertson , G. A. J. Amaratunga , J. Appl. Phys. 2004, 95, 6387.

[34] Q. Jiang , H. M. Lu , Surf. Sci. Rep. 2008, 63, 427.

[35] a) R. Koch , D. Hu , A. K. Das , Phys. Rev. Lett. 2005, 94, 146101; 15904079 10.1103/PhysRevLett.94.146101

[36] a) X.‐B. Cheng , H.‐J. Peng , J.‐Q. Huang , F. Wei , Q. Zhang , Small 2014, 10, 4257; 25074801 10.1002/smll.201401837

[37] a) C.‐M. Park , J.‐H. Kim , H. Kim , H.‐J. Sohn , Chem Soc Rev 2010, 39, 3115; 20593097 10.1039/b919877f

[38] F. Ding , Y. Liu , X. Hu , Electrochem. Solid‐State Lett. 2006, 9, A72.

[39] C. Gu , T.‐Y. Zhang , Langmuir 2008, 24, 12010. 18785717 10.1021/la802354n

[40] D. Aurbach , I. Weissman , A. Zaban , O. Chusid , Electrochim. Acta 1994, 39, 51.

[41] a) J. S. Kim , W. Y. Yoon , Electrochim. Acta 2004, 50, 531;

[42] C. Wang , D. Wang , C. Dai , J. Electrochem. Soc. 2008, 155, A390.

[43] a) Z. Liang , D. Lin , J. Zhao , Z. Lu , Y. Liu , C. Liu , Y. Lu , H. Wang , K. Yan , X. Tao , Y. Cui , Proc. Natl. Acad. Sci. USA 2016, 113, 2862; 26929378 10.1073/pnas.1518188113 PMC4801240

[44] C.‐P. Yang , Y.‐X. Yin , S.‐F. Zhang , N.‐W. Li , Y.‐G. Guo , Nat. Commun. 2015, 6, 8058. 26299379 10.1038/ncomms9058 PMC4560781

[45] M. Z. Mayers , J. W. Kaminski , T. F. Miller , J. Phys. Chem. C 2012, 116, 26214.

[46] a) Y. Lu , M. Tikekar , R. Mohanty , K. Hendrickson , L. Ma , L. A. Archer , Adv. Energy Mater. 2015, 5, 1402073;

[47] J. Zheng , P. Yan , D. Mei , M. H. Engelhard , S. S. Cartmell , B. J. Polzin , C. Wang , J.‐G. Zhang , W. Xu , Adv. Energy Mater. 2016, 6, 1502151.

[48] M. S. Whittingham , Chem. Rev. 2004, 104, 4271. 15669156 10.1021/cr020731c

[49] Y. Idota , T. Kubota , A. Matsufuji , Y. Maekawa , T. Miyasaka , Science 1997, 276, 1395.

[50] Y.‐C. Chang , H.‐J. Sohn , C.‐H. Ku , Y.‐G. Wang , Y. Korai , I. Mochida , Carbon 1999, 37, 1285.

[51] P. Poizot , S. Laruelle , S. Grugeon , L. Dupont , J. M. Tarascon , Nature 2000, 407, 496. 11028997 10.1038/35035045

[52] P. L. Taberna , S. Mitra , P. Poizot , P. Simon , J. M. Tarascon , Nat. Mater. 2006, 5, 567. 16783360 10.1038/nmat1672

[53] C. K. Chan , H. Peng , G. Liu , K. McIlwrath , X. F. Zhang , R. A. Huggins , Y. Cui , Nat. Nano 2008, 3, 31. 10.1038/nnano.2007.411 18654447

[54] H.‐X. Zhang , C. Feng , Y.‐C. Zhai , K.‐L. Jiang , Q.‐Q. Li , S.‐S. Fan , Adv. Mater. 2009, 21, 2299.

[55] S. Yang , X. Feng , S. Ivanovici , K. Müllen , Angew. Chem. Int. Ed. 2010, 49, 8408. 10.1002/anie.201003485 20836109

[56] A. Magasinski , P. Dixon , B. Hertzberg , A. Kvit , J. Ayala , G. Yushin , Nat. Mater. 2010, 9, 353. 20228818 10.1038/nmat2725

[57] Z.‐S. Wu , W. Ren , L. Xu , F. Li , H.‐M. Cheng , ACS Nano 2011, 5, 5463. 21696205 10.1021/nn2006249

[58] A. R. Armstrong , C. Lyness , P. M. Panchmatia , M. S. Islam , P. G. Bruce , Nat. Mater. 2011, 10, 223. 21317903 10.1038/nmat2967

[59] H. Wu , G. Chan , J. W. Choi , I. Ryu , Y. Yao , M. T. McDowell , S. W. Lee , A. Jackson , Y. Yang , L. Hu , Y. Cui , Nat. Nanotechnol. 2012, 7, 310. 22447161 10.1038/nnano.2012.35

[60] X. Ji , D.‐Y. Liu , D. G. Prendiville , Y. Zhang , X. Liu , G. D. Stucky , Nano Today 2012, 7, 10.

[61] L. Qie , W.‐M. Chen , Z.‐H. Wang , Q.‐G. Shao , X. Li , L.‐X. Yuan , X.‐L. Hu , W.‐X. Zhang , Y.‐H. Huang , Adv. Mater. 2012, 24, 2047. 22422374 10.1002/adma.201104634

[62] Q. Hu , Nature Outlook 2015, 526, http://www.nature.com/nature/outlook/batteries/pdf/batteries.pdf, accessed February2016.

[63] Y. Li , K. Yan , H.‐W. Lee , Z. Lu , N. Liu , Y. Cui , Nat. Energy 2016, 1, 15029.

[64] D. H. Lee , D. O. Shin , W. J. Lee , S. O. Kim , Adv. Mater. 2008, 20, 2480.

[65] X. Wang , J. Liu , Y. Wang , C. Zhao , W. Zheng , Mater. Res. Bull. 2014, 52, 89.

[66] S. Chandrashekar , N. M. Trease , H. J. Chang , L.‐S. Du , C. P. Grey , A. Jerschow , Nat. Mater. 2012, 11, 311. 22327745 10.1038/nmat3246

[67] M. Ohring , The Materials Science of Thin Films, Academic Press, San Diego, CA, USA 2001, pp. 379–383.

[68] G. N. Lewis , F. G. Keyes , J. Am. Chem. Soc. 1913, 35, 340.

[69] a) R. R. Agarwal , Technical Reports, DOE/ER/04445‐T3, Illinois Inst. of Tech, Chicago (USA) Dept. of Chemical Engineering, 1982;

[70] a) M. M. Thackeray , W. I. F. David , P. G. Bruce , J. B. Goodenough , Mater. Res. Bull. 1983, 18, 461;

[71] a) D. Aurbach , A. Zaban , A. Schechter , Y. Ein‐Eli , E. Zinigrad , B. Markovsky , J. Electrochem. Soc. 1995, 142, 2873;

[72] Y. Lu , Z. Tu , J. Shu , L. A. Archer , J. Power Sources 2015, 279, 413.

[73] a) A. A. Wheeler , B. T. Murray , R. J. Schaefer , Physica D 1993, 66, 243;

[74] X.‐B. Cheng , Q. Zhang , J. Mater. Chem. A 2015, 3, 7207.

[75] L. M. Suo , Y. S. Hu , H. Li , M. Armand , L. Q. Chen , Nat. Commun. 2013, 4, 1481. 23403582 10.1038/ncomms2513

